# Blood pressure polygenic score, cardiorespiratory fitness and odds of dementia: the HUNT Study

**DOI:** 10.1093/ageing/afag214

**Published:** 2026-07-20

**Authors:** Maren Lerfald, Karsten Øvretveit, Tom Ivar Lund Nilsen, Rannveig Sakshaug Eldholm, Nora Grøtting, Brooke N Wolford, Geir Selbaek, Linda Ernstsen

**Affiliations:** Faculty of Medicine and Health Sciences, Department of Public Health and Nursing, Norwegian University of Science and Technology, Trondheim, Norway; Clinic of Medicine, St. Olavs Hospital, Trondheim University Hospital, Trondheim, Norway; HUNT Center for Molecular and Clinical Epidemiology, Department of Public Health and Nursing, Faculty of Medicine and Health Science, Norwegian University of Science and Technology, Trondheim, Norway; Faculty of Medicine and Health Sciences, Department of Public Health and Nursing, Norwegian University of Science and Technology, Trondheim, Norway; Clinic of Emergency Medicine and Prehospital Care, St. Olavs Hospital, Trondheim University Hospital, Trondheim, Norway; Faculty of Medicine and Health Sciences, Department of Neuromedicine and Movement Science, Norwegian University of Science and Technology, Trondheim, Trøndelag, Norway; Department of Geriatrics, Clinic of Medicine, St. Olavs Hospital, Trondheim University Hospital, Trondheim, Norway; Faculty of Medicine and Health Sciences, Department of Public Health and Nursing, Norwegian University of Science and Technology, Trondheim, Norway; HUNT Center for Molecular and Clinical Epidemiology, Department of Public Health and Nursing, Faculty of Medicine and Health Science, Norwegian University of Science and Technology, Trondheim, Norway; Norwegian National Centre for Ageing and Health, Vestfold Hospital Trust, Oslo, Norway; Faculty of Medicine, University of Oslo, Oslo, Norway; Department of Geriatric Medicine, Oslo University Hospital, Oslo, Norway; Faculty of Medicine and Health Sciences, Department of Public Health and Nursing, Norwegian University of Science and Technology, Trondheim, Norway; Clinic of Medicine, St. Olavs Hospital, Trondheim University Hospital, Trondheim, Norway

**Keywords:** dementia, blood pressure, polygenic score, cardiorespiratory fitness, older people

## Abstract

High blood pressure (BP) is linked to an increased dementia risk. The role of sex and the impact of cardiorespiratory fitness (CRF) on the potential association between genetic predisposition to high blood pressure and dementia are less understood. Our aim was to investigate if there is an association between genetic predisposition to high systolic blood pressure (SBP) and occurrence of dementia in males and females and whether CRF modifies this association. This prospective cohort study utilised data from the population-based Trøndelag Health Study in Norway. We included 9145 participants ≥70 years in the HUNT4 70+ sub-study. The sex-specific association between the BP polygenic score (PGS) and dementia was estimated by logistic regression. Analyses were stratified by CRF to investigate potential effect modification by CRF. Among 5011 females (mean age 78.5 years) and 4134 males (mean age 77.4 years), 812 (16.2%) and 570 (13.8%) dementia cases were identified, respectively. Females in the highest fifth of the PGS had an increased odds of dementia [odds ratio (OR) = 1.45; 95% confidence interval (CI) 1.10 to 1.90]. This was not observed in males (OR = 1.03; 95% CI 0.77 to 1.37). The association was somewhat stronger in females with low fitness (OR = 1.53; 95% CI 1.08 to 2.17) than in females with high fitness (OR = 1.12; 95% CI 0.78 to 1.63). Genetic predisposition to high SBP was associated with higher odds of dementia in females, particularly if they had low CRF. Future studies should further examine sex-specific effects of genetic and modifiable factors on dementia risk.

## Key Points

- The sex-specific association between genetic predisposition to high systolic blood pressure and dementia is less investigated.
- Our results indicate that genetic liability to higher blood pressure is associated with higher odds of dementia in females.
- The observed association in females may be modified by cardiorespiratory fitness.
- Future research should focus on sex differences when investigating genetic and modifiable risk factors for dementia.

## Introduction

Dementia is a multifactorial disease where lifestyle and genetics have impact on disease risk [1]. There is solid evidence that hypertension in midlife increases dementia risk [2–5]. Therefore, prevention and treatment of hypertension are key to prevent and delay dementia [6–8]. While the association between hypertension in midlife and dementia is well established, few studies report sex-specific results [9]. Existing research indicates sex differences, and a systematic review found that women with higher midlife systolic blood pressure (SBP) or hypertension have a higher dementia risk compared to men with similar blood pressure (BP) traits [9].

The biology of BP is complex with a genetic architecture including over 2000 independent genetic signals encompassing thousands of single nucleotide polymorphisms identified [10]. As many genetic variants each contribute to a small proportion of the total heritability, polygenic scores (PGSs) have been developed to estimate the total genetic burden of a given disease or trait [11]. Recent research has found an association between genetic predisposition to high BP and dementia [12], and between genetic predisposition to hypertension, and worse cognitive performance [13].

Cardiorespiratory fitness (CRF) reflects the body’s ability to transport and utilise oxygen to perform physical activity and is considered a reflection of the body’s overall health [14]. High CRF is associated with reduced dementia risk [15]. As CRF is a well-established determinant for health [16], we aim to investigate the association between genetic predisposition to high SBP and occurrence of dementia in females and males separately and assess whether this association is modified by CRF.

## Methods

### Study sample

**Figure 1:**
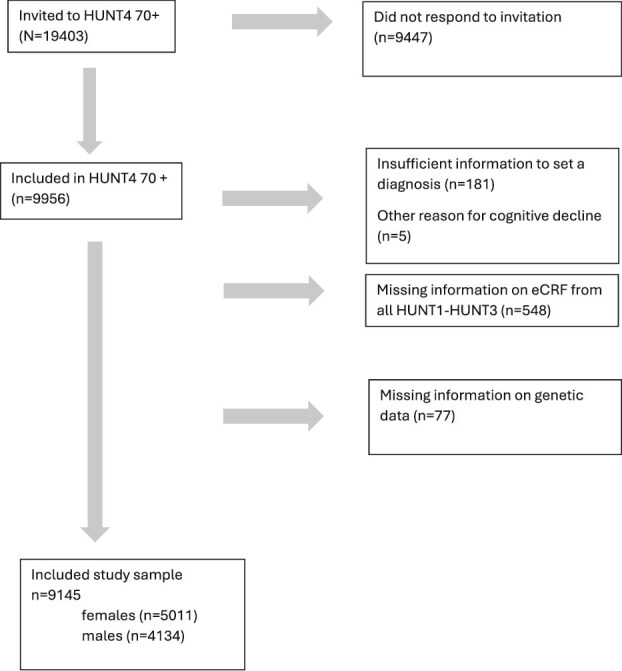
Flow chart of included study sample.

In this cohort study, we used data from the population-based Trøndelag Health Study (HUNT) in central Norway. The HUNT Study was first conducted in 1984–86 (HUNT1) with surveys roughly every 10 years with the latest in 2017–19 (HUNT4) [17–20]. The adult population has been invited to take part in assessments consisting of questionnaires, interviews, clinical examinations, laboratory measurements and providing biological samples [21, 22]. In this study, we included participants in the sub-study HUNT4 70+, which invited 19 403 inhabitants aged 70 years and older, where 9956 (51.3%) participated [23]. The HUNT4 70+ study included a cognitive assessment where participants were diagnosed with either dementia, mild cognitive impairment or no cognitive impairment [24]. We excluded individuals without sufficient information from the cognitive assessment (*n* = 181), with cognitive impairment due to depression (*n* = 5), with missing information on the estimated CRF (eCRF) variable from all HUNT1–3 (*n* = 548) and those without genetic data (*n* = 77). Finally, 4134 males and 5011 females were included (Figure 1).

### Dementia

The cognitive assessment included information from questionnaires, interviews with next-of-kin, assessment tools encompassing the areas of cognition, neuropsychiatric symptoms, start and development of symptoms, subjective cognitive decline and daily functioning [23]. Trained health personnel performed the assessment. Two physicians, from a pool of nine, with scientific and clinical expertise in either geriatrics, old-age psychiatry or neurology made diagnoses independently based on the results and followed the DSM-5 criteria [25]. Details concerning the cognitive assessment in HUNT4 70+ are presented in Gjøra *et al.* [23].

### Polygenic score associated with systolic blood pressure

To estimate genetic susceptibility to high SBP, we used a previously published PGS associated with SBP (SBP_PGS_) [26], which is available in the PGS Catalog [27] (Publication ID: PGP000772, score ID: PGS005350). Details of the PGS calculation are described in Øvretveit *et al.* [26]. Genotyping and imputation were performed as previously described [21]. Briefly, genotyping was performed using Illumina HumanCoreExome arrays. Quality control excluded samples with low call rates, contamination, chromosomal abnormalities or discrepancies between chromosomal and self-reported sex. Genotype calling followed a Genome Studio quality control protocol. Variants failing mapping, cluster separation, Hardy–Weinberg equilibrium or low call rates, were excluded. Imputation was conducted on 69 716 samples with genetics similar to reference populations of European ancestry using Minimac39 with default settings and a customised Haplotype Reference Consortium [28] release 1.1 (HRC v1.1) for autosomal and chromosome X variants. The customised reference panel incorporated low-coverage whole-genome sequences from the HUNT study and HRC v1.1 data. Imputed variants with *R*^2^ < 0.3 were excluded, resulting in over 24.9 million variants. The most probable genotypes inferred from imputation (e.g. best-guess genotypes) were used in PGS calculation.

In our main analysis, we standardised the PGS by sex. Next, we categorised the standardised SBP_PGS_ into low, moderate and high PGS strata representing the bottom, moderate second–fourth and the highest quintile, respectively.

### Estimated cardiorespiratory fitness

CRF was estimated by using validated sex-specific non-exercise algorithms [29, 30]. At HUNT1 and HUNT3, the algorithm included age, measured body mass index (BMI), measured resting heart rate (RHR) and physical activity index (PA-I) [29]. The PA-I was based on the same validated PA questionnaire at HUNT1 and HUNT3 [31], where the questions assessed frequency, duration and intensity [32]. A different validated PA questionnaire was used at HUNT2 [33]. At HUNT 2, the eCRF algorithm included age, measured waist circumference (WC), measured RHR and PA [30]. PA took on the values 0 and 1 corresponding to whether participants met the weekly PA recommendations of at least 75 min of vigorous PA or 150 min of moderate PA, or an equivalent combination [30, 34]. In this study, we included the eCRF from HUNT1 if this was available, if not, from HUNT2 and if this measure was missing, we included the measure from HUNT3.

CRF algorithm used at HUNT1 and HUNT3:

Women: 70.77—(0.244*Age)—(0.749*BMI)—(0.107*RHR) + (0.213*PA-I).

Men: 92.05—(0.327*Age)—(0.933*BMI)—(0.167*RHR) + (0.257*PA-I).

CRF algorithm used at HUNT2:

Women: 78.00—(0.297*Age)—(0.270*WC)—(0.110*RHR) + (2.674*PA)

Men: 105.91—(0.334*Age)—(0.402*WC)—(0.144*RHR) + (3.102*PA)

### Covariates

We adjusted for age and age^2^ at HUNT4 when investigating the sex-specific association between SBP_PGS_ and dementia. When investigating if eCRF could modify the potential association, we adjusted for age and age^2^ at the time eCRF was measured.

### Other variables

We present descriptive information of the study sample at HUNT4 for background information. Included information was age (years), objectively measured BP (mean of second and third measure, mmHg), BMI (kg/m^2^) and RHR (last of three measures, beats per minute), genetic status of Apolipoprotein E ε4 (ApoE4) (carrier status) self-reported smoking status (never, former daily smoker, daily smoker, occasional smoker, former occasional smoker), highest education (primary, high school, college/university <4 years, college/university ≥4 years) and current use of BP medication (yes/no). Finally, self-reported information on current or previous diseases including diabetes, myocardial infarction, heart failure, atrial fibrillation and stroke were included.

### Statistical analysis

Descriptive information was reported as means with standard deviations (SDs) for continuous variables, and as total number and percentages for categorical variables. We performed sex-specific logistic regression with dementia as outcome and the continuous standardised SBP_PGS_ as exposure adjusted for age and age^2^ at HUNT4. Next, we repeated the logistic regression with categorised SBP_PGS_. Then, we calculated absolute risk differences by applying the adjrr command in Stata [35]. Multiplicative interaction was assessed by including an interaction term between sex and the SBP_PGS_ in a logistic regression encompassing the total study sample with the same covariates, both for the standardised SBP_PGS_ and categorised SBP_PGS._ To investigate if eCRF modified the association, we standardised the eCRF within 10-year age groups and divided the sample in half; one low and one high eCRF group in the sex-specific groups. Then, we performed logistic regression by eCRF level and included age and age^2^ at the timepoint of eCRF measurement as covariates. We used Stata MP version 18.0 (StataCorp.2023 *Stata Statistical Software: Release 18*. StataCorp LLC, College Station, TC) to perform all analyses, and GraphPad Prism version 10.0 (GraphPad Software, Boston, MA) for graphics.

### Sensitivity and additional analysis

ApoE4 is the strongest genetic risk factor for dementia also associated with other cardiovascular factors [36–38]. Therefore, we expected that ApoE4 could moderate the association between SBP_PGS_ and dementia, and we tested for interaction with the SBP_PGS_ by including an interaction term between ApoE4 and SBP_PGS_ in the regression. Prior to this analysis, we excluded 11 individuals due to missing ApoE4 values. We also estimated the SBP_PGS_ without the *APOE* region. To do so, we excluded 11 variants that were 10 kilobases upstream and downstream from the *APOE* start and end position. We performed inverse probability weighting in the main analysis to account for bias due to non-participation [39]. To assess linearity, we performed sex-specific logistic regression using deciles as exposure variable.

As an additional analysis, we performed a sex-specific linear regression with the Montreal Cognitive Assessment (MoCA) assessment tool [40] as outcome and the standardised SBP_PGS_ as exposure with age and age^2^ at HUNT4 as covariates. The MoCA is mainly used as a screening tool for mild cognitive impairment and includes scores from 0 to 30 [40]. Participants with missing or invalid scores were excluded prior to this analysis (*n* = 770). We also performed multinomial regression analyses using dementia subtypes as outcome measure.

### Ethics

Participants in the HUNT Study gave informed written consent. This study was approved by the Regional Committee of Medical and Health Research Ethics in Norway (REK sør-øst D 2017/382). Storage and use of data follow the General Data Protection Regulation. Data can be obtained from a third party (HUNT databank) and is not publicly available.

## Results

In total, 5011 females with mean age 78.5 years (SD 6.7) at HUNT4 and 4134 males with mean age 77.4 years (SD 6.0) at HUNT4 were included. Descriptive statistics are presented in Table 1. Among females in the low, moderate and high PGS groups, the number of dementia cases were 142 (14.2%), 499 (16.6%) and 171 (17.1%), respectively. For males, corresponding numbers were 121 (14.6%), 330 (13.3%) and 119 (14.4%).

**Table 1:** Descriptive information of the included study sample at HUNT4.

	Males (*n* = 4134)			Females (*n* = 5011)		
	Low SBP_PGS_ ^a^	Moderate SBP_PGS_ ^a^	High SBP_PGS_ ^a^	Low SBP_PGS_ ^a^	Moderate SBP_PGS_ ^a^	High SBP_PGS_ ^a^
*N* (%)	827 (20.00)	2481 (60.01)	826 (19.98)	1003 (20.02)	3006 (59.99)	1002 (20.00)
Age (years), mean (SD)	77.50 (6.06)	77.49 (6.06)	77.20 (5.97)	78.65 (6.93)	78.51 (6.72)	78.22 (6.64)
SBP (mmHg), mean (SD)	133.73 (18.70)	138.95 (19.44)	141.28 (19.14)	136.64 (21.13)	140.12 (20.75)	144.17 (20.31)
DBP (mmHg), mean (SD)	73.10 (9.99)	75.04 (10.54)	75.17 (10.00)	70.62 (9.74)	71.58 (9.82)	72.88 (9.60)
BMI (kg/m^2^), mean (SD)	27.15 (3.93)	27.35 (3.81)	27.21 (3.89)	27.01 (4.81)	27.21 (4.92)	26.91 (4.77)
RHR (beats/min), mean (SD)	69.96 (13.63)	69.83 (13.55)	70.69 (14.22)	74.06 (13.84)	74.23 (12.91)	73.46 (12.70)
ApoE4 carrier, *n* (%)	237 (28.69)	758 (30.60)	261 (31.60)	272 (27.15)	907 (30.2)	299 (29.90)
Smoking status, *n* (%)						
Never	246 (31.58)	784 (33.09)	270 (34.01)	374 (40.35)	1249 (45.37)	404 (44.10)
Former daily	459 (58.92)	1393 (58.80)	451 (56.80)	424 (45.74)	1186 (43.08)	392 (42.79)
Daily	43 (5.52)	105 (4.43)	51 (6.42)	61 (6.58)	188 (6.83)	68 (7.42)
Occasional	1 (0.13)	8 (0.34)	3 (0.38)	2 (0.22)	9 (0.33)	2 (0.22)
Former occasional	30 (3.85)	79 (3.33)	19 (2.39)	66 (7.12)	121 (4.40)	50 (5.46)
Education, *n* (%)						
Primary	152 (19.54)	500 (21.20)	175 (22.01)	323 (35.07)	1007 (36.68)	342 (37.62)
High school	376 (48.33)	1144 (48.52)	391 (49.18)	390 (42.35)	1161 (42.30)	401 (44.11)
College/Uni <4 years	144 (18.51)	393 (16.67)	118 (14.84)	110 (11.94)	349 (12.71)	86 (9.46)
College/Uni ≥4 years	106 (13.62)	321 (13.61)	111 (13.96)	98 (10.64)	228 (8.31)	80 (8.80)
Diabetes, *n* (%)	90 (11.64)	310 (13.47)	125 (15.96)	78 (8.53)	292 (10.85)	108 (11.99)
Use of BP medication, *n* (%)	269 (47.36)	1240 (67.35)	552 (80.00)	307 (46.44)	1396 (65.11)	630 (80.05)
Myocardial infarction, *n* (%)	110 (14.71)	364 (16.46)	141 (18.80)	38 (4.43)	146 (5.84)	72 (8.55)
Heart failure, *n* (%)	51 (6.92)	152 (7.00)	58 (8.01)	32 (3.73)	129 (5.20)	47 (5.66)
Atrial fibrillation, *n* (%)	108 (14.71)	373 (17.26)	163 (22.21)	85 (10.06)	260 (10.55)	101 (12.24)
Stroke, *n* (%)	67 (9.07)	230 (10.45)	83 (11.32)	71 (8.32)	219 (8.82)	85 (10.22)

^a^Categories defined according to quintiles of the SBP_PGS_ distribution: low = lowest fifth; moderate = second–fourth fifth; high = highest fifth. DBP, diastolic blood pressure.

Number with missing data on descriptive variables: Age 0, SBP 119, DBP 119, BMI 389, RHR 1303, APOE4 11, Smoking 607, Education 639, Diabetes 780, Use of BP medication 2454, Myocardial infarction 1233, Heart failure 1346, Atrial fibrillation 1381, Stroke 1303.

**Figure 2:**
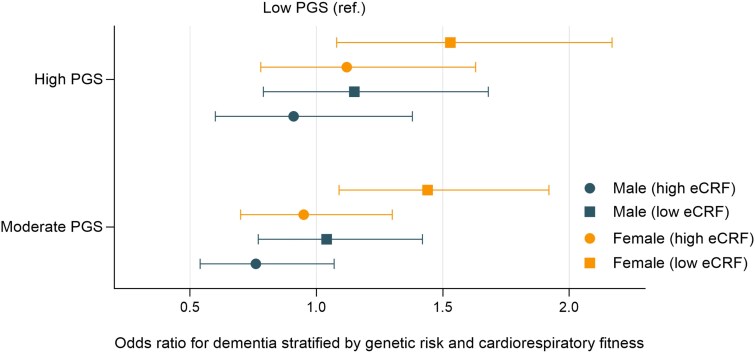
OR for dementia stratified by PGS (low: bottom quintile, moderate: second—fourth quintile, high: top quintile), and CRF level (low: bottom half, high: top half).

An association was observed between the SBP_PGS_ and dementia in females {Table 2, odds ratio (OR) per 1 SD = 1.13 [95% confidence interval (CI) 1.04–1.23]}, but not in males [OR per 1 SD = 1.01 (95% CI 0.92–1.11)]. Females in the highest quintile of the SBP_PGS_ had an OR of dementia of 1.45 (95% CI 1.10–1.90) compared to those in the lowest quintile. Corresponding numbers in males were OR = 1.03 (95% CI 0.77–1.37). Interaction test between sex and the standardised SBP_PGS_ yielded a *P*-value of .096. For the categorical SBP_PGS_, we observed evidence of statistical interaction (*P*-value = .025). When stratifying by eCRF level among females, the OR for dementia in the high PGS/high eCRF strata was 1.12 (95% CI 0.78–1.63) and OR in the high PGS/low eCRF was 1.53 (95% CI 1.08–2.17), where the low SBP_PGS_ served as the reference (see Figure 2 and Supplementary Appendix 1). Similar results were found for the PGS without the *APOE* region in females (Supplementary Appendix 2, Supplementary Appendix 3). The interaction test between ApoE4 and the SBP_PGS_ in females revealed no support for effect modification. Inverse probability weighting gave similar results as our main analysis. The full decile-based estimates are presented in Supplementary Appendix 4. The additional analysis using the MoCA score as outcome, showed significant association in females, but not in males (Supplementary Appendix 5). In the diagnosis-specific analysis, strongest association was found for vascular dementia in females, relative risk ratio = 1.55 (95% CI 1.22–1.97) (Table 3).

**Table 2:** Sex-specific adjusted OR and absolute risk difference with 95% CI for dementia by PGS for SBP.

	Males (*n* = 4134)			Females (*n* = 5011)		
	Dementia, *n* (%)	OR^a^, (95% CI)	Absolute risk difference^a^, %, (95% CI)	Dementia, *n* (%)	OR^a^ (95% CI)	Absolute risk difference^a^, %, (95% CI)
Continuous SBP_PGS_, per 1 SD^a^	570 (13.79)	1.01 (0.92, 1.11)	0.09 (−0.92, 1.10)	812 (16.20)	1.13 (1.04, 1.23)	1.40 (0.43,2.36)
Categories of SBP_PGS_^b^						
Low	121 (14.63)	Ref.	Ref.	142 (14.16)	Ref.	Ref.
Moderate	330 (13.30)	0.88 (0.70,1.12)	−1.33 (−3.92, 1.26)	499 (16.60)	1.31 (1.05, 1.65)	2.86 (0.59, 5.12)
High	119 (14.41)	1.03 (0.77, 1.37)	0.28 (−2.95, 3.52)	171 (17.07)	1.45 (1.10, 1.90)	3.94 (1.07, 6.82)

^a^Adjusted for age and age^2^ at HUNT4.

^b^Categories defined according to quintiles of the SBP_PGS_ distribution: low = lowest fifth; moderate = second–fourth fifth; high = highest fifth.

**Table 3:** Sex specific RRR of dementia with 95% CI where the continuous standardised SBP_PGS_ serve as the exposure.

Dementia diagnosis	Females			Males		
	RRR^a^	95% CI	*n* (%)	RRR^a^	95% CI	*n* (%)
No dementia (ref.)			4199 (83.8)			3564 (86.2)
AD	1.11	1.00, 1.22	479 (9.6)	0.99	0.87, 1.12	312 (7.6%)
VaD	1.55	1.22, 1.97	69 (1.4)	0.96	0.76, 1.22	73 (1.8%)
Mixed dementias	1.12	0.89, 1.41	73 (1.5)	1.11	0.83, 1.50	47 (1.1%)
Unspecified/Other	1.06	0.92, 1.23	191 (3.8)	1.05	0.88, 1.25	138 (3.3%)

^a^Adjusted for age and age^2^ at HUNT.

AD, Alzheimer’s Disease; VaD, vascular dementia; RRR, relative risk ratio.

## Discussion

An association between the SBP_PGS_ and dementia was observed in females but not in males. When stratifying by eCRF level, the point estimate was attenuated and statistically imprecise among females with higher eCRF. This indicates that CRF might modify the association in females.

A study investigating the association between polygenic predisposition to elevated BP and dementia suggested that genetically elevated BP is associated with higher dementia risk [12], however, sex-specific associations were not reported. Another study investigating the association between polygenic susceptibility to hypertension and cognitive performance concluded that a higher PGS was associated with worse cognitive performance [13]. Interaction analysis with sex did not support effect modification [13]. Our findings, on the other hand, indicate that there might be sex differences in the association between SBP_PGS_ and dementia. Previous research has identified sex differences in the genetic risk for hypertension, where the association with hypertension appears stronger in females [41, 42]. This corresponds well with research that has investigated the sex-specific association between objectively measured BP and dementia [9]. For the SBP_PGS_ used in our study, similar sex differences have been noted, however, the sample size was too small to detect significant differences [26].

Even if our results indicate sex differences, other alternative explanations should be considered. As men have a higher risk of cardiovascular events and cardiovascular mortality at an earlier age compared to women [43], this might attenuate a potential association more in males. A recent meta-analysis found no sex difference in dementia incidence globally but highlighted that life expectancy and differences in education opportunities may explain variability in differences according to sex [44]. However, it has been noted that cardiovascular risk factors are associated with greater dementia risk in females compared to males, emphasising the already established sex difference in cardiovascular risk factors [45].

There might be shared genetics among SBP_PGS,_ and genes linked to dementia or CRF. As ApoE4 is identified as a pleiotropic locus associated with outcomes related to BP, cardiovascular diseases and Alzheimer’s disease [38], we expected a possible interaction between ApoE4 and the SBP_PGS_. However, no interaction was identified and excluding the *APOE* region from the SBP_PGS_ gave similar results, indicating that other genetic exposures than ApoE4 drives the association. However, lack of statistical power or effects of competing risk related to cardiovascular outcomes can attenuate a potential interaction, meaning that a greater proportion of those with highest genetic risk for cardiovascular diseases and hypertension may be more affected by related diseases and death. Additionally, it is likely that the SBP_PGS_ and CRF have shared genetics as the SBP_PGS_ includes genetic variants from several loci also associated with lifestyle exposure [38], and a previous study found a high PGS for CRF to be associated with lower hypertension risk [46].

A strong positive association between SBP_PGS_ and vascular dementia in females was observed. The association between hypertension, cerebral small vessel disease and cognitive decline is well established and emerging evidence suggests that this relationship may be stronger in women [47]. Thus, our findings might indicate a potential sex-specific effect of SBP-related pathways on vascular dementia. However, these findings should be carefully interpreted and require confirmation in larger studies.

As genetic information might identify people at higher risk for a specific disease from birth, this gives new opportunities for intervention on modifiable risk factors before unfavourable health traits develop. This can be beneficial for prevention of several diseases related to hypertension, such as cardiovascular diseases and dementia, and is relevant for future disease prevention. However, the manner in which PGS should be used in clinical settings is under discussion [48]. One considerations is the risk of giving a wrong impression of genetic determinism, which consequently can impact one’s health behaviour negatively [48]. This is supported by a prior study investigating public knowledge on the influence of modifiable risk factors for dementia, where they found the knowledge to be low in the general population [49]. Others highlight that PGS alone does not affect health behaviour [50]. This challenge applies to communication of risk factors in general [50]. Accordingly, PGS is likely to have main benefits in clinical areas of disease risk classification and early detection, rather than promoting health behaviour changes [50].

Whether modifiable risk factors can attenuate genetic risk is a current focus in public health. A recent study found a mitigating effect of high CRF on the association between genetic predisposition for dementia and dementia development, indicating that CRF can moderate unfavourable genetic traits [51]. However, another study found that among those with high genetic dementia risk, protective associations of modifiable risk factors were not observed [52]. Recent results from the HUNT Study identified an interaction between SBP_PGS_ and eCRF with possibly greater effect among individuals with higher PGS [53]. These findings indicate that more studies are needed on the effects of modifiable risk factors among individuals with high genetic risk for dementia, or high genetic risk for traits associated with dementia.

### Strengths and limitations

Our study has several strengths, including a large study sample, where all individuals participated in a cognitive assessment with validated instruments resulting in a clinical diagnosis set by a physician. We had genetic information on the majority of participants in HUNT4 70+. Using SBP_PGS_ as exposure gives complementary information to the established association between objectively measured BP in midlife and dementia [2, 54]. The SBP_PGS_ was validated in the HUNT Study, and the derivation used repeated BP measurements in a large cohort and compared novel and traditional methods [26]. We accounted for selection bias due to non-participation by inverse probability weighting using previously published weights for the HUNT4 70+ cohort. However, several limitations should be considered. Survival bias and the effect of competing risk could affect the strength of the association between the SBP_PGS_ and dementia. Participants in HUNT4 70+, included individuals over or equal to 70 years, increasing the risk of survival bias. It is likely that those with higher SBP_PGS_ are at greater risk of cardiovascular diseases and mortality, and it is also likely that men are more affected by competing mortality given the previously mentioned sex differences in cardiovascular disease. However, in this case, the association would appear weaker than the true estimate, and even weaker in males. Our analysis suggest interaction between sex and the SBP_PGS,_ however, interaction on the OR scale should be interpreted with caution, as non-collapsibility may produce apparent differences even in the absence of true modification [55]. Additionally, stratification by sex does not fully address potential confounding from other interactions, which might result in residual bias. Since we could not confirm that dementia cases were incident, the potential for reverse causation cannot be ruled out. For the development of the SBP_PGS_, different instruments for measuring BP were used at different HUNT surveys [26]. In addition, BP medication was self-reported, where those who reported use, had 15 and 10 mmHg adjustments added to their SBP and diastolic BP, respectively, to control for pharmacological effects [26]. The SBP_PGS_ should ideally have been sex-specific, as previous studies have identified sex differences in PGS’s for SBP and hypertension [41, 42]. However, the genetic effect estimates used to derive the PGS were not estimated in a sex-specific manner and the sample size in the original study validating this PGS was too small to validate sex-specific scores [26]. Finally, CRF was not objectively measured, but algorithms have been shown to be good alternatives when objective measures are inconvenient [14].

In summary, a higher SBP_PGS_ was associated with higher odds of dementia in females, and the observed association in females might be modified by CRF. These associations need to be further explored in future research.

## Supplementary Material

aa-26-0767-File004_afag214

## Research Data Transparency and Availability

Data can be obtained from a third party (HUNT databank) and is not available to the public.
