# Supplementary material for: Blood pressure polygenic score, cardiorespiratory fitness and odds of dementia: the HUNT Study
Source: Age Ageing. 2026 Jul 20;55(7):afag214. doi: 10.1093/ageing/afag214 (PMC13384636; doi:10.1093/ageing/afag214)
Supplement: aa-26-0767-File004_afag214 [file aa-26-0767-file004_afag214.docx]

Blood pressure polygenic score, cardiorespiratory fitness and odds of dementia: the HUNT Study

*Supplementary content*

Contents

[Appendix1 2](#_Toc230611226)

[Appendix 2 3](#_Toc230611227)

[Appendix 3 3](#_Toc230611228)

[Appendix 4 4](#_Toc230611229)

[Appendix 5a 5](#_Toc230611230)

[Appendix 5b 5](#_Toc230611231)

# Appendix1

Odds ratio (OR) with 95% confidence interval (CI) for dementia in the different polygenic score (PGS) categories, stratified by sex and by high or low estimated cardiorespiratory fitness (eCRF) level.

| **PGS category** | **Sex** | **eCRF** | **OR** | **95% CI** | **Sample, n** | **Dementia, n (%)** |
| --- | --- | --- | --- | --- | --- | --- |
| Low | Male | High |  |  | 416 | 55 (13%) |
| Low | Male | Low |  |  | 411 | 66 (16%) |
| Low | Female | High |  |  | 498 | 67 (13%) |
| Low | Female | Low |  |  | 505 | 75 (15%) |
| Middle | Male | High | 0.76 | 0.54-1.07 | 1234 | 129 (10%) |
| Middle | Male | Low | 1.04 | 0.77-1.42 | 1247 | 201 (16%) |
| Middle | Female | High | 0.95 | 0.70-1.30 | 1474 | 195 (13%) |
| Middle | Female | Low | 1.44 | 1.09-1.92 | 1532 | 304 (20%) |
| High | Male | High | 0.91 | 0.60-1.38 | 417 | 51 (12%) |
| High | Male | Low | 1.15 | 0.79-1.68 | 409 | 68 (17%) |
| High | Female | High | 1.12 | 0.78-1.63 | 453 | 80 (15%) |
| High | Female | Low | 1.53 | 1.08-2.17 | 469 | 91 (19%) |

# Appendix 2

Odds ratio and 95% confidence interval for dementia stratified by polygenic score for systolic blood pressure (not including *APOE*)

|  | **Males (n=4134)** | | **Females (n=5011)** | |
| --- | --- | --- | --- | --- |
|  | OR^b^ (95% CI) | Absolute risk difference^b^, % | OR^b^ (95% CI) | Absolute risk difference^b^, % |
| Continuous SBP_PGS_, per SD | 1.00 (0.91, 1.10) | -0.01 (-1.02, 1.00) | 1.14 (1.05, 1.24) | 1.49 (0.52, 2.47) |
| Catergories of SBP_PGS_^a^ |  |  |  |  |
| Low | Ref. | Ref. | Ref. | Ref. |
| Moderate | 0.87 (0.69, 1.10) | -1.48 (-4.07, 1.12) | 1.27 (1.01, 1.59) | 2.47 (0.21, 4.73) |
| High | 0.96 (0.72, 1.29) | -0.43 (-3.66, 2.80) | 1.54 (1.18, 2.02) | 4.74 (1.85, 7.63) |

Abbreviations: SBP_PGS_ = polygenic score for systolic blood pressure; OR = odds ratio; CI = confidence interval; SD = standard deviation

^a^ Categories defined according to quintiles of the SBP_PGS_ distribution: low = lowest fifth; moderate = 2^nd^-4^th^ fifth; high = highest fifth.

^b^Adjusted for age and age^2^ at HUNT4

# Appendix 3

Odds ratio and 95% confidence interval for dementia stratified by polygenic score for systolic blood pressure (not including *APOE*) and by cardiorespiratory fitness level in females

|  | **Females (n=5011)** | |
| --- | --- | --- |
|  | Low eCRF | High eCRF |
|  | OR^b^ (95% CI) | OR^b^ (95% CI) |
| Categories of SBP_PGS_^a^ |  |  |
| Low | Ref. | Ref. |
| Moderate | 1.37 (1.03, 1.82) | 0.95 (0.70, 1.31) |
| High | 1.73 (1.23, 2.44) | 1.17 (0.81, 1.69) |

Abbreviations: SBP_PGS_ = polygenic score for systolic blood pressure; OR = odds ratio; CI = confidence interval; SD = standard deviation

^a^ Categories defined according to quintiles of the SBP_PGS_ distribution: low = lowest fifth; moderate = 2^nd^-4^th^ fifth; high = highest fifth.

^b^Adjusted for age and age^2^ at HUNT4

# Appendix 4

Odds ratio of dementia by PGS deciles. Adjusted for age and age^2^ at HUNT4.

| **PGS deciles** | **Females** | | **Males** | |
| --- | --- | --- | --- | --- |
|  | OR | 95% CI | OR | 95% CI |
| Ref. |  |  |  |  |
| 2 | 1.28 | 0.86, 1.91 | 0.99 | 0.66, 1.49 |
| 3 | 1.53 | 1.04, 2.26 | 0.82 | 0.54, 1.24 |
| 4 | 1.23 | 0.83, 1.81 | 1.08 | 0.72, 1.62 |
| 5 | 1.30 | 0.88, 1.93 | 0.82 | 0.53, 1.25 |
| 6 | 1.80 | 1.23, 2.61 | 0.82 | 0.53, 1.24 |
| 7 | 1.47 | 0.99, 2.18 | 0.95 | 0.63, 1.43 |
| 8 | 1.65 | 1.12, 2.42 | 0.80 | 0.52, 1.23 |
| 9 | 1.48 | 1.00, 2.19 | 0.97 | 0.64, 1.47 |
| 10 | 1.80 | 1.23, 2.63 | 1.07 | 0.71, 1.61 |

# Appendix 5a

Regression results for MoCA-score as outcome and the continuous (z-score) polygenic score as exposure among females. Adjusted for age and age^2^ at HUNT4.

|  | **Mean difference** | **Standard error** | **t-statistics** | **P > \|t\|** | **95% CI** |
| --- | --- | --- | --- | --- | --- |
| **Continuous SBP_PGS_** | -0.190 | 0.061 | -3.11 | 0.002 | -0.310, -0.070 |
| **Age** | 1.439 | 0.222 | 6.47 | 0.000 | 1.003, 1.875 |
| **Age^2^** | -0.011 | 0.001 | -8.17 | 0.000 | -0.014, 0.009 |
|  | | | | | |
| **R^2^** | 0.2529 | | | | |
| **Number of observations** | 4530 | | | | |
| **Mean MoCA (SD)** | 22.937 (4.756) | | | | |

# Appendix 5b

Regression results for MoCA-score as outcome and the continuous (z-score) polygenic score as exposure in males. Adjusted for age and age^2^ at HUNT4.

|  | **Mean difference** | **Standard error** | **t-statistics** | **P > \|t\|** | **95% CI** |
| --- | --- | --- | --- | --- | --- |
| **Continuous SBP_PGS_, per SD** | -0.098 | 0.064 | -1.52 | 0.128 | -0.224, 0.028 |
| **Age** | 1.229 | 0.250 | 4.91 | 0.000 | 0.739, 1.719 |
| **Age^2^** | -0.010 | 9.971 | -6.11 | 0.000 | -0.013, -0.006 |
|  | | | | | |
| **R^2^** | 0.1639 | | | | |
| **Number of observations** | 3845 | | | | |
| **Mean MoCA (SD)** | 22.561 (4.359) | | | | |

Abbreviations: SBP_PGS_ = polygenic score for systolic blood pressure; CI = confidence interval; SD = standard deviation
